# Works in Progress

**Published:** 1842-06

**Authors:** 


### Works in Progress.

Dr. James D. M'Cabe, of Richmond, Va. is, we understand, preparing a work on the *Institutes of Dental Surgery,* in which he intends to present a broad view of the science in all its *phases* and *details.* A work of this description is much needed, and we are gratified to learn, that one so competent to the task, has undertaken its preparation. Dr. McCabe is a good writer, and we think we hazard little in saying, that his work will be an interesting one; and one that will meet with a ready and quick sale. This is perhaps, saying a great deal in advance, but from our knowledge of the abilities of Dr. M. we have confidence to believe that our expectations will be fully realized. We hope that it will not be long before we shall have an opportunity of announcing its publication.

*Traité complet de l'Art du Dentiste, d'après l'état actuel des connaissances.* Par F. Maury, dentiste de l'Ecole Royale Polytechnic. Nouvelle edition, avec un Atlas de 40 planches. Paris, 1833, 8o. pp. 578.

*A Complete Treatise on the Dental Art, based upon actual experience.* By F. Maury, Dentist of the Royal Polytechnic School. New edition, with an Atlas of 40 plates. Paris, 1833; 8vo. pp. 578.

The above comprehensive and excellent treatise on Dental Surgery, is, we are happy to inform our readers, about to be translated, by Joseph B. Savier, D. D. S. of Baltimore; and that those of our professional brethren who have not had an opportunity of seeing it, may be able to form a correct idea of the plan of the treatise, we would state, that as its title implies, it embraces the various branches that properly appertain to the province of the dentist, and we know of no other of the same extent (578 pages) that contains the same amount of useful matter.

Dr. Savier, though young in the profession, bids fair to become one of its brightest ornaments, and the performance of the task in which he is now engaged, will entitle him to the warmest thanks of his professional brethren.

The work is divided into three parts. The first contains an "Anatomical description of the teeth and their appendages; the phenomena of first and second dentition; the diseases of the dental organs proper and those of the mouth separately." The second part is devoted to "hygiene and therapeutics of the mouth at the various periods of life, operations that belong essentially to the dental art, and the various instruments suited to each operation." The third part embraces the "Mechanical department of the art, the mode of supplying lost teeth; definition of technicalities employed in the art, and an alphabetical table of most of the authors, who during the last two centuries and upwards, have written upon some department of the profession."

That the work is held in high estimation by the dental profession in France, is proven by the fact, that it has passed through, we believe, five editions within the last fifteen or twenty years.

